# Matrix- and Technology-Dependent Stability and Bioaccessibility of Strawberry Anthocyanins during Storage

**DOI:** 10.3390/antiox10010030

**Published:** 2020-12-30

**Authors:** Anna-Sophie Stübler, Lena Böhmker, Andreas Juadjur, Volker Heinz, Cornelia Rauh, Avi Shpigelman, Kemal Aganovic

**Affiliations:** 1 DIL German Institute of Food Technologies e.V., Prof.-v.-Klitzing-Str. 7, 49610 Quakenbrück, Germany; lena.boehmker@web.de (L.B.); a.juadjur@dil-ev.de (A.J.); v.heinz@dil-ev.de (V.H.); 2 Fachgebiet Lebensmittelbiotechnologie und–prozesstechnik, Technische Universität Berlin, Königin-Luise-Straße 22, 14195 Berlin, Germany; cornelia.rauh@tu-berlin.de; 3 Faculty of Biotechnology & Food Engineering, Technion—Israel Institute of Technology, Haifa 32300, Israel; avis@bfe.technion.ac.il

**Keywords:** anthocyanins, pulsed electric fields (PEF), high-pressure processing (HPP), storage, bioaccessibility

## Abstract

Anthocyanins are often associated with health benefits. They readily degrade during processing and storage but are also dependent on the matrix conditions. This study investigated how strawberry anthocyanins are affected by preservation technologies and a relatively protein-rich kale juice addition during storage. A strawberry–kale mix was compared to a strawberry–water mix (1:2 wt; pH 4), untreated, thermally, pulsed electric fields (PEF) and high-pressure processing (HPP) treated, and evaluated for anthocyanin stability and bioaccessibility during refrigerated storage. The degradation of strawberry anthocyanins during storage followed first-order kinetics and was dependent on the juice system, preservation technology and anthocyanin structure. Generally, the degradation rate was higher for the strawberry–kale mix compared to the strawberry–water mix. The untreated sample showed the highest degradation rate, followed by HPP, PEF and, then thermal. The relative anthocyanin bioaccessibility after gastric digestion was 10% higher for the thermally and PEF treated samples. Anthocyanin bioaccessibility after intestinal digestion was low due to instability at a neutral pH, especially for the strawberry–kale mix, and after thermal treatment. The storage period did not influence the relative bioaccessibility; yet, the absolute content of bioaccessible anthocyanins was decreased after storage. This research further presents that processing and formulation strongly affect the stability and bioaccessibility of anthocyanins during storage.

## 1. Introduction

Anthocyanins are polyphenolic compounds and an important pigment of many fruits and vegetables. They are reported to exhibit health-promoting properties, reducing the risk for cardiovascular diseases, cancer and diabetes by their capacity to scavenge free radicals and indirectly inhibit cell proliferation [1]. Strawberries are a major source of anthocyanins in human nutrition, with pelargonidin-3-O-glucoside as the main compound, accounting for 77–90% of the total anthocyanins [2]. However, anthocyanins are unstable and readily degrade and undergo oxidation or polymerization reactions during processing and storage due to their chemical structure. The occurrence and rate of these reactions are influenced by the presence of oxidative enzymes such as polyphenoloxidase or peroxidase and other food compounds (e.g., vitamin c and fructose), pH value, exposure to light and high temperature [3,4,5,6]. 

Due to increasing consumer demand for products with potential health effects and minimally processed products, more complex juices, such as blends and smoothies with considerable amounts of anthocyanins, are developed and, in some cases, on the market, preserved by alternative technologies such as pulsed electric fields (PEF) and high-pressure processing (HPP). Thus, adjusting the formulation and applying preservation technologies working on a different principle than heat, such as pulsed electric fields (PEF) and high-pressure processing (HPP), can be named as two possible approaches to enhance anthocyanin retention during processing and storage.

PEF is based on the effects of the electroporation of bacterial cells, whereas HPP is based on the changes in morphology and subcellular structures, as well as biochemical, physiological and genetic alterations leading to microbial inactivation [7,8,9]. Anthocyanins degrade at high temperatures, as seen after the pasteurization of strawberries (90 °C for 5 min), resulting in a 16% decrease of pelargonidin-3-O-glucoside and 5% for pelargonidin-3-O-rutinoside [10]. Generally, HPP leads to a less pronounced degradation of anthocyanins compared to the thermal treatment, as reported for acai, fermented pomegranate and strawberries [11,12,13]. Similar results were reported for strawberry puree and blood orange juice, in which no significant changes in anthocyanins after the HPP treatment (400–600 MPa, 15 min) compared to the untreated sample were found, while the thermal treatment (70 °C, 2 min) of the strawberry puree resulted in a significant decrease by 28%, suggested to be caused by thermal or enzymatic degradation [14,15]. The PEF treatment of strawberry juice at different processing conditions (20–35 kV/cm, 100–2000 µs) resulted in retention above 96% [16]. The comparison of the PEF (35 kV/cm, 1700 µs) and thermal treatment (90 °C, 30 or 60 s) on strawberry juice revealed a slight but significant anthocyanin decrease after both treatments [17].

Formulation is another approach that might alter anthocyanin stability, as anthocyanins are likely to interact with other food components. These interactions and their responses to alternative processing technologies and during storage are, so far, not extensively investigated. Only a few studies have evaluated the additional effects of different matrices or matrix components, such as proteins, on the stability of anthocyanins in complex multicomponent products. Yet, even fewer studies have evaluated the effects of different processing techniques on storage. 

Anthocyanin-rich black currant juice was mixed with apple, persimmon and peach juice, where faster anthocyanin degradation during storage at 4 °C was observed in the mixed juice compared to the single black currant juice, while thermal pasteurization had no major effects [18]. Another study investigated the effects of preservation on the stability and bioaccessibility of compounds such as vitamin C, phenolics and carotenoids in juice blends (orange, kiwi, pineapple and mango) and formulations with cow milk and soymilk. The study reported an overall improved quality of PEF and HPP-treated juice/soymilk beverages compared to the thermally treated one and a reduced bioaccessibility of hydrophilic compounds in milk/juice beverages [19,20]. In our previous work, it was already observed that mixing strawberry puree and kale juice results in interactions and decreased concentrations of free anthocyanins and polyphenols, while the processing technologies had a less pronounced impact [21,22]. Studies on anthocyanin interactions are mainly performed in model systems, and the named interactions often represent a strategy to modify the stability of anthocyanins during processing and storage. A model beverage system based on black carrot extract exhibited increased storage stability of anthocyanins after the addition of different proteins, especially denatured whey protein isolate. The improved stability was suggested to be the outcome of an anthocyanin–protein complex formation via hydrogen bonding [23]. In line with these findings, the addition of amino acids and peptides to such model beverages increased the color stability of the contained anthocyanins during storage [24]. 

The degradation of anthocyanins during storage was also investigated in various fruit products and model systems and was reported to follow first-order kinetics, with increased degradation at higher storage temperatures [25,26,27,28]. The anthocyanin loss of HPP-treated strawberry juice (600 MPa, 4 min) during six-months of storage exhibited an increased degradation rate for cloudy, compared to clear, strawberry juice. It was suggested to be caused by oxygen absorbed by the pulp particles [29]. Generally, an increase in the pressure level showed an accelerating effect on anthocyanin degradation during storage [15,30,31]. A similar degradation rate was reported during refrigerated storage at 4 °C for HPP-treated and untreated bayberry juice [31]. However, in blood orange juice, a decreased degradation rate for HPP-treated samples compared to untreated samples was observed during refrigerated storage. The inactivation of oxidative enzymes related to food quality, such as peroxidase and polyphenoloxidase, is less effective with HPP than with thermal treatment, often leading to accelerated degradation in HPP-treated compared to thermally treated samples [13,32]. After PEF (electric field strength 35 kV/cm for 1700 µs) or thermal treatment (90 °C for 30 or 60 s), significant anthocyanin degradation occurred in strawberry juice during storage at 4 °C and was slightly more pronounced in the thermal samples compared to PEF [17]. However, in a model system, the PEF treatment showed no significant influence on the degradation rate of cyanidin-3-O-glucoside during refrigerated storage [33]. To the best of our knowledge, the effect of alternative processing on complex multicomponent juice systems during storage was not yet investigated.

Along with anthocyanins’ stability during processing and storage, their claimed health effects likely also require their stability, availability and absorption during gastrointestinal digestion. Such properties are also influenced by the processing technology, matrix properties and interactions and storage conditions [34]. However, the bioaccessibility of anthocyanins, as assessed after in-vitro digestion, was assumed to be low when compared to other polyphenols. This is probably due to the chemical structure and pH sensitivity of anthocyanins [35,36]. From the processing aspect, the PEF treatment (25 kV/cm, 32–256 kJ/kg) of a papaya–mango juice led to no significant changes in the total anthocyanin content during gastrointestinal digestion compared to the untreated sample, except for the intense PEF treatment resulting in significantly higher values after intestinal digestion [37]. The bioaccessibility of anthocyanins in HPP-treated strawberry extract was similar to the one of the untreated sample [34]. The bioaccessibility of anthocyanins in sour cherry was investigated as influenced by different dairy matrices, and no effect was observed after intestinal digestion, while soymilk increased the anthocyanin bioaccessibility after gastric digestion [38]. In a similar study, the effects of the food matrix (milk, soymilk and water) and processing on the bioaccessibility of phenolics in a fruit juice blend were investigated. The bioaccessibility of phenolic compounds was reduced due to the effects of milk [39]. Unfortunately, there is only limited literature data with regards to anthocyanin bioaccessibility as effected by storage, processing and formulation.

The current study aimed to investigate the stability of anthocyanins in strawberry puree during refrigerated storage, as influenced by preservation technologies, including PEF, HPP and a thermal treatment, and by formulation with a complex matrix being relatively protein-rich—kale juice. Furthermore, the bioaccessibility was assessed before and after storage for a more comprehensive understanding of the potential health effects of such complex multicomponent juice systems. 

## 2. Materials and Methods 

### 2.1. Raw Materials and Chemicals

Individually Quick-Frozen (IQF) strawberries (*Fragaria Ananassa* “Senga Sengana”) from Poland were purchased from Fruity King BV (Barendrecht, The Netherlands) and stored at −20 °C before the processing. Freshly harvested, washed kale (*Brassica oleracea* L. var. *sabellica*) was purchased from ELO-frost GmbH & Co. KG (Vechta, Germany) and instantly processed into juice. The juice was stored at −20 °C before processing. Chemicals were of analytical grade.

### 2.2. Preparation of Juice Formulation

Strawberry puree was prepared with a vacuum bowl cutter (Kilia Ltd., Birmingham, UK). Kale juice was prepared with a shredder (Model BG2-5, 5 kW, voran Maschinen GmbH, Pichl bei Wels, Austria) and a packing press with a rotary cage (Model 100P2, voran Maschinen GmbH, Pichl bei Wels, Austria). Collected juice was frozen at −20 °C until further usage. Strawberries and kale juice were defrosted overnight at room temperature. To produce the multicomponent juices, strawberry puree and kale juice were mixed manually in a 1:1 ratio (wt). Appropriate controls without the interfering matrix of strawberry–water and kale–water were prepared. To eliminate the pH value effect on the anthocyanin stability, pH values of strawberry-water and kale-water were adjusted to the pH of the strawberry-kale mix (4.0 ± 0.1) using 12-M HCl or NaOH, respectively. 

Total soluble solids, protein content and sugar level of the untreated juice formulations are shown in Table 1. As these values were neither significantly changed by the different processing conditions nor by storage (data not shown), the values are only presented for the untreated juice directly after preparation. 

### 2.3. Processing Equipment and Conditions

Thermal treatment, serving as a reference for a conventional preservation step, was performed at 72 °C at a flowrate of 35 L/h, resulting in a holding time of 1 min. A tube-in-tube heat exchanger (DIL e.V., Quakenbrück, Germany) was used for thermal treatment but, also, for preheating before and cooling after the PEF treatment. The PEF treatment was conducted using a pilot system (HVP 5 kW, DIL e.V., Quakenbrück, Germany) connected to the tube-in-tube heat exchanger. After preheating to 35 °C, the product was PEF-treated at an electric field strength of 11.7 kV/cm and an energy input of 120 kJ/kg. Bipolar rectangular pulses were applied with a pulse width of 20 µs in two successive colinear chambers with an electrode gap of 10 mm. The HPP treatment was conducted on a small industrial unit (wave 6000/55, Hiperbaric S.A., Burgos, Spain) at 600 MPa for 1 min at room temperature. The processing conditions were selected based on comparable microbial inactivation resulting in a 5log reduction, as determined in the preliminary experiments, and were considered in an industrially relevant range [22].

### 2.4. Sample Handling and Storage

Samples were filled in 30-mL PET bottles (Nipak BV, Lopik, The Netherlands) that were previously rinsed with 70% ethanol under a laminar flow cabinet. For the shelf life tests, the samples were stored at 4 °C in the dark. Samples were taken at day 0 (after sample production) and after 3, 7, 14, 21, 28 and 42 days. The samples were directly frozen at −40 °C until further analyses. Analyzed samples are untreated (control), thermal, PEF and HPP-treated strawberry–water system (S), strawberry–kale mix (M) and kale–water system (K). 

### 2.5. In-Vitro Digestion for Bioaccessibility

Semi-dynamic in-vitro digestion was conducted on a dual auto-titration unit (Titrando 902, Metrohm, Herisau, Switzerland), with a double-wall reactor based on the harmonized protocol of Minekus et al. (2014), as previously described [21,40]. Samples were taken after 0 (G0), 30 (G30), 60 (G60), 63 (D63), 90 (D90) and 120 (D120) min. The sample at digestion time point 0 contained no digestive enzymes. Samples were immediately frozen in liquid nitrogen and stored at −40 °C until further analyses. The in-vitro digestion was done for samples directly after processing (day 0) and at the end of the investigated refrigerated storage (day 42) for the thermal, PEF and HPP-treated samples. Due to microbial instability, the untreated sample was not considered at the end of the storage. 

### 2.6. Microbiological, Chemical and Physical Analyses 

#### 2.6.1. Microbiological Quality

The microbiological quality was assessed by analyzing the total aerobic plate counts, total yeasts and total molds, according to the methods described in DIN EN ISO 4833-2:2013 and ASU L 01.00-37. For the total aerobic plate count, the samples were cultivated on a nonselective solid plate count agar medium and incubated for 72 h at 30 °C. For yeast and molds, the sample was cultivated on a yeast extract-glucose-chloramphenicol agar at 25 °C for 4 days. The colonies were counted and expressed as the number of colonies forming units per ml of sample.

#### 2.6.2. Residual Enzyme Activity Measurements of Peroxidase (POD) and Polyphenoloxidase (PPO)

The residual enzyme activity of POD and PPO were measured based on the method of Yue-Ming, Zauberman, and Fuchs (1997) [41]. Both methods include enzyme extraction, followed by mixing of the enzyme extract with a defined poly-/phenolic solution and photometrically quantified oxidation. Enzymes were extracted by mixing 5 g of the sample with 25-mL potassium citrate buffer (100 mM, pH 4) containing 0.5-g polyvinylpyrrolidin for absorption/removal of interfering polyphenols. The mixture was homogenized with an Ultraturrax at 35,000 rpm, incubated for 30 min on ice and further centrifuged at 15,000× *g* for 20 min at 4 °C. The protein content was measured according to the Bradford assay, with a calibration curve of bovine serum albumin by mixing 3 mL of the Bradford reagent with 0.1 mL of the extracted sample. This was incubated for 5 min at room temperature and measured at 595 nm.

For the PPO activity, the diluted extract was mixed (ratio 1:40 vol) with 25-mM 4-methylcatechol in potassium phosphate buffer (100 mM, pH 6.8) and immediately measured at 410 nm for 2 min with 10-s intervals. For the POD activity, the diluted extract was mixed (ratio 1:60 vol) with 0.1% H_2_O_2_ and 0.2% guaiacol in potassium phosphate buffer (100 mM, pH 6.8) and immediately measured at 470 nm for 4 min with 10-s intervals.

The enzymatic activities were then calculated using the slope of the curve showing the absorbance increase and the molar absorption coefficient of 1010 M^−1^cm^−1^ for 4-methylcatechol and 26.600 M^−1^cm^−1^ 3,3″-dimethoxy-4,4′-biphenoquinon. The activity was calculated as the relative residual activity compared to the untreated strawberry-kale mix and expressed in %. Measurements were done using a UV/Vis spectrophotometer (Spectro 40, Analytik Jena AG, Jena, Germany). 

#### 2.6.3. Color Determination (CIE-L*a*b*)

Color and its stability during storage is a major quality indicator of food products by consumers. The color was measured using a spectrophotometer (CM-5, Konica Minolta Business Solution GmbH, Langenhagen, Germany), and data were reported in the CIE L*a*b* colorimetric system. A constant sample volume was placed in the glass cuvette and measured. The lightness L*, red (+) and green (-) amount a* and the -blue (-) and yellow (+) amount b* were obtained, and the total color difference ΔE was calculated as follows:

(1) $$\Delta E_{1,2}=\;\sqrt{{{(L}_{1}^{*}{-L}_{2}^{*})}^{2}{+(a}_{1}^{*}{-a}_{2}^{*}{)}^{2}{+\;(b}_{1}^{*}{-b}_{2}^{*}{)}^{2}}$$

The total color difference ΔE can be classified as follows: not noticeable 0–0.5, slightly noticeable 0.5–1.5, noticeable 1.5–3.0, well visible 3.0–6.0 and great >6.0. Values above 3.0 are considered significantly different but might still be acceptable [42,43,44].

#### 2.6.4. Ascorbic Acid/Dehydroascorbic Acid

Dehydro-/ascorbic acid was determined according to the method of Kneifel and Sommer (1985) for all samples after processing (day 0) and after storage (day 42) [45]. The sample was homogenized with a m-phosphoric acid-acetic acid solution and further mixed with double-distilled water. Activated charcoal was added to the sample to transform *L*-ascorbic acid to dehydro-*L*-ascorbic acid and well-mixed, followed by filtration and dilution with water and a sodium-acetate solution. This solution was again mixed with a phenylenediamine solution and incubated for 16 h in the dark. After membrane filtration, the samples were injected and separated during high performance liquid chromatography (HPLC Alliance Waters Separations Modul 2695, Waters, Milford, MA, USA) equipped with a column LiChrospher 100 RP and detected by a fluorescence detector ((Fluorescence Detector 2475 Multi Lambda, Waters, Milford, MA, USA).

#### 2.6.5. Antioxidant Capacity (ORAC)

The antioxidant capacity was determined using the oxygen radical absorbance capacity (ORAC) method, as previously described [21], by a microplate reader (Synergy H1, BioTek Instruments Inc., Winooski, VT, USA). The sample was incubated with 8-nM fluorescein in phosphate buffer (75 mM, pH 7.4) at 37 °C, 75 mM AAPH was added and the absorbance was recorded each minute for 1 h.

#### 2.6.6. Total Monomeric Anthocyanin Content 

The total monomeric anthocyanin content (MAC) was determined by the pH differential method, as described by Stübler et al. (2020) [22]. Samples were centrifuged at 25,830× *g* for 20 min at 4 °C. The supernatant of the sample was further diluted 10-fold in 0.025-M potassium chloride buffer at pH 1 and 0.4-M sodium acetate buffer at pH 4.5. The absorbance was measured at 520 nm and 700 nm, with a microplate reader (Infinite^®^ 200Pro, Tecan Group Ltd., Männedorf, Switzerland). The MAC is calculated as depicted below and expressed in mg pelargonidin-3-O-glucoside per liter:

(2) $$\mathrm{MAC}\;\left(\frac{\mathrm{mg}\;P3G}{l}\right)=\frac{({\left(A_{520}-A_{700}\right)}^{\mathrm{pH}\;1}-{{(A}_{520}-A_{700})}^{\mathrm{pH}\;4.5})\cdot \mathrm{MW}\cdot \mathrm{DF}\cdot 10^{3}}{\varepsilon \cdot l}$$

with the absorbance A, the molecular weight MW of pelargonidin-3-O-glucoside (433.4 $\frac{g}{\mathrm{mol}}$), the dilution factor DF, the path length l and the molar absorptivity ε of 22,400 $\frac{l}{\mathrm{mol}\cdot \mathrm{cm}}$.

#### 2.6.7. Anthocyanin Kinetics 

Specific anthocyanins originating from strawberries were analyzed according to Juadjur and Winterhalter (2012) and Stübler et al. (2020), with slight modifications [22,46]. Analyses were performed on a Nexera-i LC-2040C 3D Plus (Shimadzu Corporation, Kyoto, Japan) equipped with a UV/Vis detector and a 3-μm Luna^®^ column C18(2) (4.6 mm × 250 mm) (Phenomenex, Torrance, CA, USA). Identification of single anthocyanins was previously performed and described [22]. To determine anthocyanins in the aqueous phase during storage, samples were centrifuged at 15,000× *g* for 30 min at 4 °C and filtered using 0.45 µm before injection in the system.

The degradation of the anthocyanins during refrigerated storage was then modelled using first-order kinetics with SigmaPlot 14.0 (Systat Software Inc., San Jose, CA, USA). This is described by Equation (3) (*c*—relative concentration, *c_0_*—relative concentration at time 0, *k*—degradation constant and *t*—time in days). The fitting of the model was appropriate with the coefficient of determination (R²) of 0.9 and higher for all samples.

(3) $${c\;=\;c}_{0}\cdot e^{-kt}$$

#### 2.6.8. Relative Bioaccessibility of Anthocyanins 

The bioaccessibility was assessed as described in Stübler et al. (2020) with slight modifications, using an Amicon^®^ filter with a 10-kDa membrane [22]. The digested sample was centrifuged for 20 min at 21,380× *g* to eliminate large particles possibly clogging the filter unit. The supernatant was centrifuged for 60 min at 14,000× *g* at 20 °C in the filter unit, and the permeate was collected. The residues in the filter were removed, and a washing step using a mixture of water and ethanol (1:1 vol) was applied to collect the anthocyanins trapped by the filter membrane. The washing included another centrifugation step of 30 min at 14,000× *g* at 4 °C. The permeate was collected, diluted 10-fold, filtered using a 0.45-µm membrane filter and injected into the high performace liquid chromatography equipped with a diode array detector (HPLC-DAD). It was calculated as the ratio of the area under the curve (AUC) of the respective samples at a certain digestion point to the AUC in extracted untreated strawberry water at the respective storage point (Equation (4)).

(4) $$\mathrm{BA}\;=\;\frac{{\mathrm{AUC}}_{\mathrm{Digestion}\;\mathrm{time}}}{{\mathrm{AUC}}_{\mathrm{Absolute}\;\mathrm{of}\;\mathrm{untreated}\;\mathrm{strawberry-water}}}$$

For the anthocyanin extraction, the sample was homogenized at 20,000 rpm for 1 min and mixed at a ratio of 1:1 (vol) with 70% aqueous methanol containing 1% formic acid. After vortexing, the samples were placed for 15 min in the ultrasound bath, further diluted by 2, filtered using a 0.45-µm membrane filter and injected.

### 2.7. Statistical Analyses 

In-vitro digestion experiments were done in duplicate; all other analyses were at least in triplicate. Results are presented as means ± standard deviation and analyzed using analysis of variance (one- and two-way ANOVA) followed by Tukey’s multiple comparison test (*p* < 0.05) using SigmaPlot 14.0 (Systat Software Inc., San Jose, CA, USA). Furthermore, a Pearson correlation was conducted with the named software.

## 3. Results and Discussion

### 3.1. Microbiological Quality

**Figure 1:**
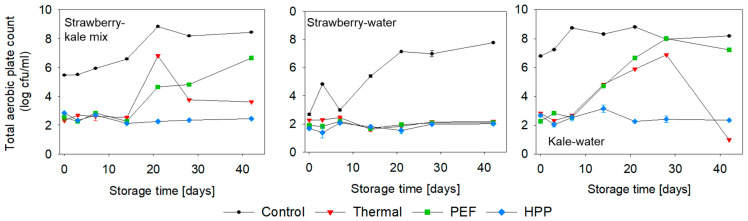
Total aerobic plate count (log CFU/mL) during refrigerated storage at 4 °C (n = 2). PEF: pulsed electric fields and HPP: high-pressure processing.

For assessing the microbial quality of the samples, the total aerobic plate count and yeast and molds were tested during storage. As seen in Figure 1, the microbial contamination mostly derived from the kale matrix, which is shown by high microbial counts in the untreated kale-water sample. The preservation step led to a comparable but statistically insignificantly different microbial inactivation of 3log in the strawberry-kale samples, regardless of the technology. In the kale-water samples, a microbial reduction by 4log was achieved, while, in the strawberry-water samples, the microbial inactivation was 1log, likely due to the low initial contamination. During storage, the HPP-treated strawberry-kale mix and kale-water mix were microbiologically stable, while the thermally and PEF-treated named mixes exhibited outliers or an increase in the total aerobic plate count during storage. This could be related to the possible recontamination during the manual filling of the samples after PEF and thermal treatments, while the HPP was applied to the samples in the package. The strawberry-water mix was stable during storage, regardless of the treatment, due to a very low initial microbial contamination and likely less favorable growing conditions. Yeast and molds were below the detection limit regardless of the formulation, processing technology and storage time.

### 3.2. Residual Enzyme Activity of Peroxidase (POD) and Polyphenoloxidase (PPO)

**Figure 2:**
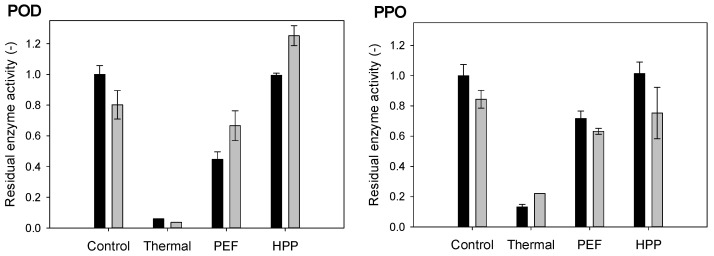
Residual enzyme activity of peroxidase (POD) and polyphenoloxidase (PPO) in the strawberry-kale mix (compared to the untreated sample before storage) immediately after the treatment (black) and after storage (grey).

**Figure 3:**
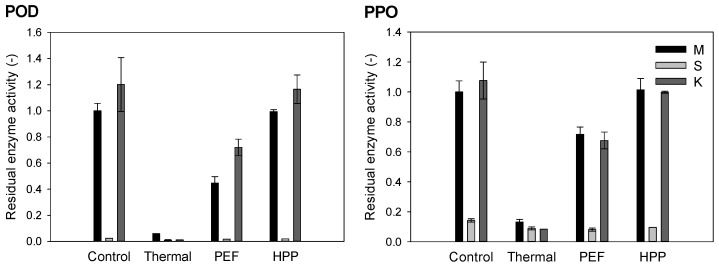
Residual enzyme activity of POD and PPO in the strawberry-kale mix (M), strawberry-water mix (S) and kale-water mix (K) (compared to the untreated strawberry-kale mix) after processing.

The activity of naturally occurring POD and PPO leads to the degradation of valuable compounds, e.g., polyphenols in fruits and vegetables. To preserve the stability, nutritional quality and fresh character during storage, low or no enzyme activity of POD and PPO, among others, is desired. The residual enzymatic activity of the investigated samples was majorly derived from kale, while strawberries contributed minorly to the activity of POD and PPO. As expected and depicted in Figure 2 and Figure 3, thermal processing led to the highest enzyme inactivation, with a relative residual activity of 6.0% ± 0.0% for POD and 13.2% ± 1.7% for PPO in the strawberry–kale mix, followed by a PEF with residual activities of 44.7% ± 4.9% for POD and 71.7% ± 4.9% for PPO. The HPP led to no enzyme inactivation compared to the untreated strawberry–kale mix. During storage, the relative activity of POD in the PEF and HPP-treated mixes slightly increased, while it decreased for PPO. 

Compared to other studies, a decrease in the POD and PPO activity to 70% and 55% of the initial activity, respectively, was observed in kale puree after thermal treatment at 70 °C for 2 min [44], while a thermal treatment at 72 °C for 15 s of apple juice, 90 °C for 15 min of strawberry puree and 90 °C for 60 s of broccoli juice resulted in (almost) complete inactivation of POD and PPO [13,47,48]. A study further compared low and high-intensity PEF treatments (12 kV/cm, 80 and 130 kJ/kg) of apple juice, leading to the partial inactivation of POD and PPO for the low PEF treatment (comparable to the observations in our results) and a higher inactivation for the high-intensity PEF treatment, suggested to be related to the thermal impact occurring due to dissipating energy as a result of Joule heating during the PEF treatment [47,49]. Another study of enzyme activity in strawberry puree reported only 3% residual PPO activity after high-intensity PEF at 35 kV/cm, a frequency of 229 Hz, a pulse width of 3.2 and 4.2 µs, treatment time of 1500 µs and bipolar pulses [50], while the minimal residual activity in broccoli juice reached 36% after high-intensity PEF (35 kV/cm, 986 kJ/m³, treatment time 2000 µs, bipolar pulse), while lower intensities of PEF (15 kV/cm, 1932 kJ/m³, treatment time 500 µs, monopolar) even triggered the PPO activity in broccoli juice [48].

In terms of the pressure impact on POD and PPO activity, the HPP at 600 MPa for 3 min did not result in significant changes of the POD and PPO activity in apple juice [47], similar to our results, while the HPP of strawberry puree at 500 MPa for 15 min at 50 °C led to a partial inactivation, resulting in residual activities of 50% for POD and 72% for PPO [13], which, in terms of the treatment intensity, is significantly higher than the conditions used in our current study. 

Different results can be found in the literature on the impact of storage on the enzyme activity. The results on PEF and HPP-treated apple juice showed a decrease in the enzyme activity for PPO and POD; however, when the residual enzyme activity was low after the treatment, as for a thermal or a high-intense PEF treatment, no effect of storage was observed [47], while both increased in acidified vegetables [51]. The current results support previous studies that indicate that enzyme inactivation by different processing technologies and their activity during storage is a matrix (source, variety, etc.) and processing and intensity-dependent, with the thermal effect being, in many cases, the most effective in enzyme inactivation [52].

### 3.3. Ascorbic Acid/Dehydroascorbic Acid 

Ascorbic acid/dehydroascorbic acid is a quality-determining factor of fruit products and, also, reported to affect the stability of anthocyanins, especially during storage [53]. The concentration of ascorbic acid/dehydroascorbic acid was measured before and after 42 days of refrigerated storage in the differently processed samples (Table 2). No significant differences were observed between the strawberry and the strawberry–kale mix before and after treatment, regardless of the technology. In the kale–water system, the concentration was below the detection limit. This demonstrated that detectable ascorbic acid/dehydroascorbic acid was largely derived from strawberries in the current system. As related to kale, it is generally known to be rich in vitamin C; however, it is often bound to indole-3-carbinol and present as an ascorbigen [54,55]. Furthermore, it was reported that ascorbigens exhibit the highest stability at pH 4, which is the pH value in the investigated juice systems [56]. Significantly higher concentrations of ascorbic acid/dehydroascorbic acid were detected for the thermally treated strawberry-kale mix before and after storage, while the untreated, HPP and PEF-treated samples exhibited comparable concentrations of ascorbic acid/dehydroascorbic acid. In the strawberry–water mix, a minor effect of processing on ascorbic acid/dehydroascorbic acid concentrations was observed. The thermally treated strawberry–water mix resulted in the highest concentration of ascorbic acid/dehydroascorbic acid but was only significantly different when compared to the HPP-treated strawberry–water mix. After the storage, all treated samples exhibited a significant decrease in concentration by 60–70%. Other studies on the thermal treatment of kale reported a substantial loss of vitamin C in the range of 84% [44], while a different study in apple juice reported a loss of only 10% after PEF and thermal treatment. Only HPP-treated apple juice (600 MPa, 3 min) showed a more significant decrease [47]. In the current study, the increased ascorbic acid/dehydroascorbic acid concentrations after thermal treatment compared to the untreated were unexpected, as ascorbic acid/dehydroascorbic acid is known to be unstable at exposure to high temperatures. It might be related to an increased extraction from the matrix due to the thermal treatment. Another study on the thermal treatment (85 °C, 2 min) and HPP (400–600 MPa, 3 min) of strawberry puree reported similar results: a slight yet not significant increase after thermal treatment and a significant decrease after HPP at 400 MPa was observed for vitamin C [57]. However, a study on the impact of HPP on vitamin C in strawberry juice showed no significant influence of pressure at intensities between 400–600 MPa for 15 min, while the results of thermal treatments in these studies showed more pronounced degradation (21%) [14]. In blueberry juice, mango nectar and strawberry juice, no or only minor effects of the PEF treatment on the vitamin C concentration was reported, while the thermal treatment led to the enhanced degradation of vitamin C [17,58,59]. Similarly, no significant difference after the PEF treatment was observed in apple juice [60]. To conclude, this study showed a minor effect of the PEF and HPP, yet a slight increase in ascorbic acid/dehydroascorbic acid concentrations after the thermal treatment when compared to the untreated samples. However, the major decrease in ascorbic acid/dehydroascorbic acid concentrations was observed after storage due to degradation regardless of the preservation treatment and formulation.

### 3.4. Color Difference Dependent on Processing during Storage 

Color is an important quality-determining factor for fruit and vegetable products that can influence consumer acceptance and can trigger purchase [31]. It strongly depends on the initial quality, way of processing and storage conditions. Color changes occurring during storage are mostly related to chemical reactions, such as polyphenol degradation [61]. The current results (ΔE values) show that processing led to a significant color modification in all juice systems (Table 3). Thermal and PEF processing resulted in the largest color differences, followed by HPP, when compared to the untreated sample. For the strawberry–water mix, the PEF-treated sample was most divergent, while, for the strawberry–kale mix and kale–water mix, the thermally treated samples were the most divergent. 

Aligned with our results, in strawberry puree, the ΔE was significantly larger after the thermal treatment (70 °C, 2 min) compared to HPP (600 MPa, 15 min) [14]. The PEF-treated strawberry juice showed a higher color difference compared to the thermally treated juice [17]. Another study reported the color of strawberry to be less heat-stable at pH 5 or higher than the natural pH of strawberry at ~3.7 [62]. This effect was intentionally mitigated in this study by adjusting the pH of all systems to the pH value of 4.0 (Section 2.2). 

The color difference ΔE during storage increased significantly in the strawberry-kale mix, especially for the untreated control sample, followed by the HPP and then PEF (Table 4). The thermally treated strawberry–kale mix exhibited the lowest changes in color differences, with the maximum values around two, indicating color stability during storage, probably due to the low activity of oxidative enzymes in those samples (Section 3.2).

Previously, an increase in color difference during storage was observed in strawberries [63]. The storage of pasteurized strawberries led to a decrease in the a* value, resulting in a decreased red color, which is aligned with the observations in our strawberry–water mix [26]. However, the strawberry–kale mix showed a slight increase in the a* value (data not shown). The a* value expresses the color along the axis from green (negative value) to red (positive value). Thus, in the strawberry–kale mix, an overlying effect of a decrease in redness but, similarly, a decrease in green color might occur during storage. This might indicate the degradation of the green and red color pigments. A study of different thermal treatments of kale puree resulted in a significant increase in the L* and a* values [44], explaining the increase in a* in the strawberry–kale mix. The decrease in the a* value for the strawberries and increase in the a* value for kale (data not shown) can be related to the degradation of anthocyanins and chlorophylls that are responsible for the red and green colors. Furthermore, the obtained results indicate that the color differences in the strawberry-kale mixes during storage are higher in samples with high residual enzyme activity (Section 3.2), resulting in the degradation of valuable color responsible components, including anthocyanins and other polyphenols, during storage. 

### 3.5. Antioxidant Capacity

**Figure 4:**
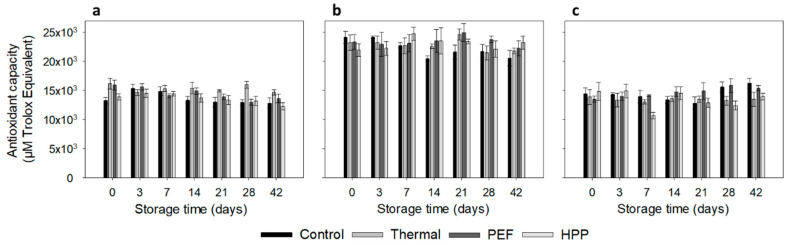
Antioxidant capacity via ORAC (µM Trolox Equivalent) for strawberry–water (**a**), strawberry–kale mix (**b**) and kale–water mix (**c**) during storage at 4 °C (n = 3).

The antioxidant capacity determined by ORAC was higher in the strawberry–kale mix compared to strawberry–water and kale–water (Figure 4). This is a result of the high antioxidant capacity of each separate matrix of strawberry or kale; thus, when combined in the strawberry–kale mix, the antioxidant capacity is significantly increased compared to the diluted 50% systems: strawberry–water and kale–water. However, the total antioxidant capacity in the strawberry–kale mix is not additive, and it is partly masked by the interaction of the strawberry and the kale matrices [64]. The antioxidant capacity remained constant during refrigerated storage, with minor changes in the dependence of processing technologies and storage time, as also previously observed in several other studies [22,65,66]. Overlaying effects can occur together, with the formation of new components from degradation having similar antioxidative capacities. However, several studies observed a decrease in the antioxidant capacity during storage measured by different methods in a HPP-treated and thermally treated strawberry puree, strawberry–milk beverage and smoothies [63,67,68].

### 3.6. Anthocyanin Degradation Kinetics during Storage

**Figure 5:**
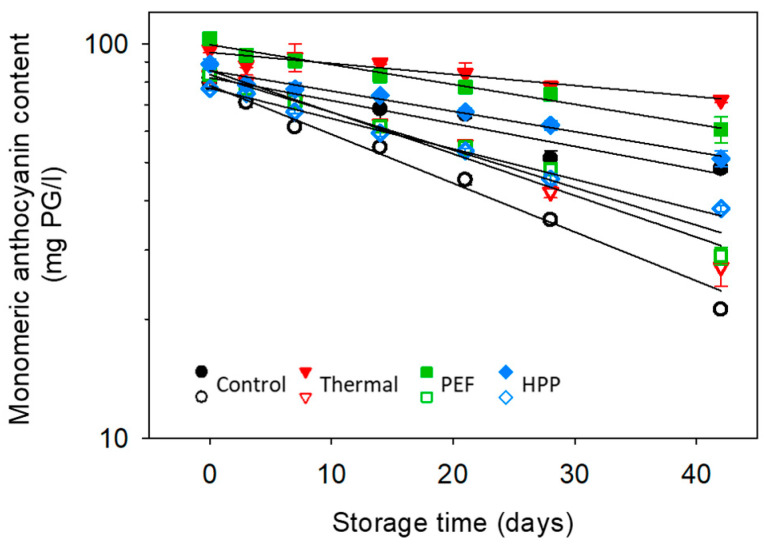
Kinetic modelling of the monomeric anthocyanin contents (pH differential) during refrigerated storage at 4 °C for the strawberry–kale mix (empty) and strawberry–water (full) (n = 3).

**Figure 6:**
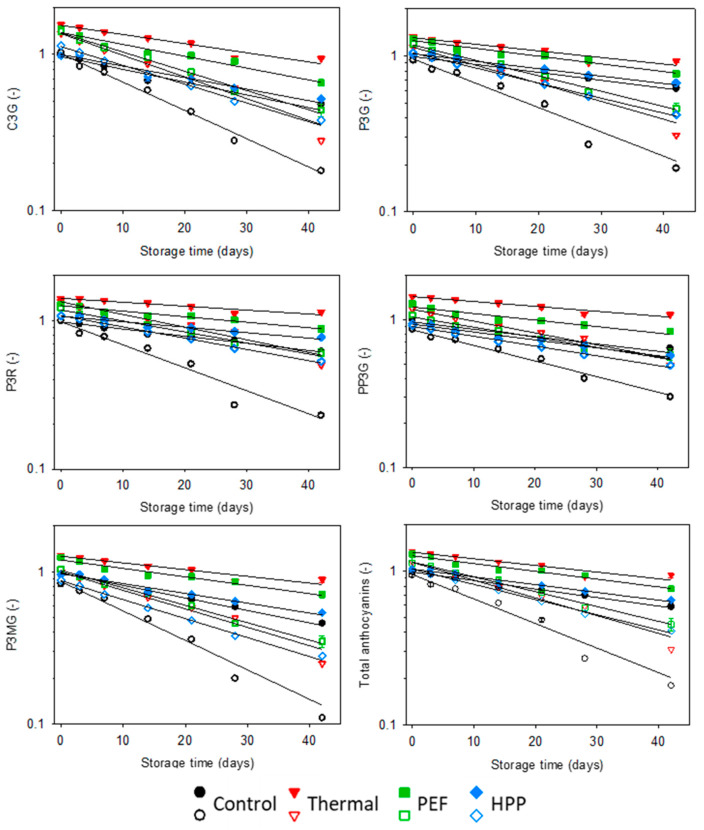
Kinetic modelling for the relative anthocyanin contents in the aqueous phase measured via HPLC-DAD during refrigerated storage at 4 °C for the strawberry-kale mix (empty) and strawberry-water (filled) (n = 3).

The major anthocyanins detected in strawberry-containing samples are pelargonidin-3-O-glucoside (PG), accounting for 80% of the total anthocyanin content, followed by pelargonidin-3-O-malonylglucoside (PMG) with 12%, cyanidin-3-O-glucoside (CG) with 5.4% and pelargonidin-3-O-rutinoside (PR) and 5-pyrano-pelargonidin-3-O-glucoside (PPG), each with 1.3%. The degradation kinetics of these major strawberry anthocyanins, as well as the total monomeric anthocyanin contents (MAC) in the aqueous phase in the strawberry–water and strawberry–kale mix, followed a first-order degradation model. The kinetics of anthocyanins during storage are displayed in Figure 5 and Figure 6 and their characteristic values in Table 5.

The total anthocyanins, as a sum of all anthocyanins chromatographically determined, showed high correlations with the total monomeric anthocyanins, as determined using the pH differential method (0.960) and PG as the major anthocyanin from the strawberries (0.997), as previously reported [69]. 

When interpreting the kinetics and the degradation constant *k*, differences between the juice systems, processing technologies and the type of anthocyanin were observed. The degradation constant was significantly higher for the strawberry–kale mix compared to the strawberry–water mix, meaning that anthocyanins are less stable in the complex strawberry-kale mix. The significant difference between the formulations might be at least partially caused by the substantially higher activity of oxidative enzymes in the strawberry–kale mix compared to the strawberry–water mix (Section 3.2) [15]. Previously, the anthocyanin stability was also reported to be strongly influenced by the surrounding matrix, including the pH and chemical composition [27]. In our case, the effect of the pH in the formulations was eliminated. Further explanations could be related to the possibly increased oxygen absorbed by the pulp particles in the strawberry–kale mix leading to enhanced anthocyanin degradation, as previously observed in cloudy compared to clear strawberry juice [29], or metal ions present in the kale juice accelerating the oxidation of anthocyanins [70]. In addition, all investigated preservation technologies slowed down the degradation of the anthocyanins in the aqueous phase, while the untreated samples exhibited the highest degradation rate during the refrigerated storage. In the strawberry–water mix, the thermal treatment resulted in the slowest degradation along with the storage (lowest *k*), followed by the PEF and HPP. This directly relates to the oxidative enzyme activities in the strawberry–water mix. In contrast to the reported results in the strawberry–water mix, the degradation in the strawberry–kale mix was faster for the thermal compared to PEF and HPP, which are in a comparable range. Higher degradation rates in the thermally treated strawberry–kale mix can be considered surprising, as the oxidative enzymes were largely inactivated, while the enzyme activity in the HPP-treated strawberry–kale mix was more similar to the untreated sample. Generally, the literature reports lower degradation rate constants during storage for thermally treated juice systems, while HPP-treated juices showed comparable or slightly lower degradation rate constants compared to the untreated juices [71,72], as observed for the strawberry–water mix. However, Aaby et al. (2018) reported that processing, including thermal and HPP, has no significant effect on the anthocyanin stability, while storage (6 °C during 49 days) was the major parameter significantly influencing the total monomeric anthocyanin content [57].

A limited amount of scientific data is available on anthocyanin degradation in complex multicomponent systems after a preservation treatment with thermal, PEF or HPP. A possible reason for increased degradation in the thermally treated strawberry–kale mix could be the increased polymerization during storage [27]. The results of our current study indicate that the effects of formulation, including kale, can also influence the processing effects. Thus, interactions between the formulation and processing effects are suggested. The HPP and PEF resulted in improved anthocyanin stability in the strawberry–kale mix, while it was contrary for the strawberry–water system. However, it must be considered in the interpretation of the results that only the soluble fraction of anthocyanins was assessed.

As already mentioned, the degradation kinetics can be influenced by the anthocyanin structure. PMG exhibited slightly decreased stability during storage compared to PG, except for the thermally treated samples, where the oxidative enzymes were largely inactivated by the treatment. Previously, the malonyl moiety was reported to decrease the stability of cyanidin-MG, compared to CG in blood orange juice [69]. The degradation of PMG is initiated by the cleavage of the malonyl group, resulting in the formation of PG, which is reported to occur faster than the degradation of PG. Thus, the degradation rate of PG might be underestimated due to this overlaying effect of the degradation of anthocyanins based on the structure of PG. From the results, CG exhibited an increased degradation constant in all systems, regardless of the processing, and, therefore, faster degradation compared to PG (Figure 6). This was suggested to be a result of the increased reactivity of CG due to the additional hydroxyl group at the B-ring [27]. PR exhibited a slight but statistically significant decrease in *k*-values, except for the untreated samples, which was previously observed in model and juice systems due to the larger disaccharide moiety and possible steric hindrance compared to monosaccharides [5,6,18,28,73,74]. However, in another study, the enhanced thermal stability of CG compared to CR in a black rice bran colorant powder was observed [75]. Yet, Wilkes et al. (2014) reported that the sugar type had only a minor effect on the stability of anthocyanins during long-term storage, yet were more important during processing [76]. As determined with two-way ANOVA, PPG exhibited, overall, the highest stability, which might be caused by two interfering effects—firstly, the high stability of pyrano-anthocyanins in general and, secondly, the storage of the juice leading to the formation of newly formed PPG from monomeric PG and low molecular compounds [77,78,79].

As anthocyanins are colored pigments, they could be correlated to certain color parameters, such as the a* value. Significant Pearson correlations (*p* < 0.05) were found positively for all strawberry–water samples, while negatively for the strawberry–kale samples, except for thermally treated samples in any of the analyzed systems (Table 6). This is aligned with previous observations in color (Section 3.4) and the anthocyanin degradation in strawberries related to a decrease in the anthocyanin pigments. However, in the strawberry–kale mix, the effect was counterbalanced by the chlorophyll degradation resulting in a decreased green color.

### 3.7. Relative Bioaccessibility (BA) of Anthocyanins before and after Storage

Bioaccessibility is defined as the fraction being released and made available for absorption in the gastrointestinal tract [80]. Some studies suggest the absorption of anthocyanins taking place already during gastric digestion [81]. The bioaccessibility can be calculated and presented in various ways. To maximize the possibility to conclude, regarding the impacts of processing and formulation, on the bioaccessibility, in this section, it will be referred to as a gastric and intestinal bioaccessibility relative to the anthocyanin content of the extracted untreated strawberry–water sample at the respective storage time.

**Figure 7:**
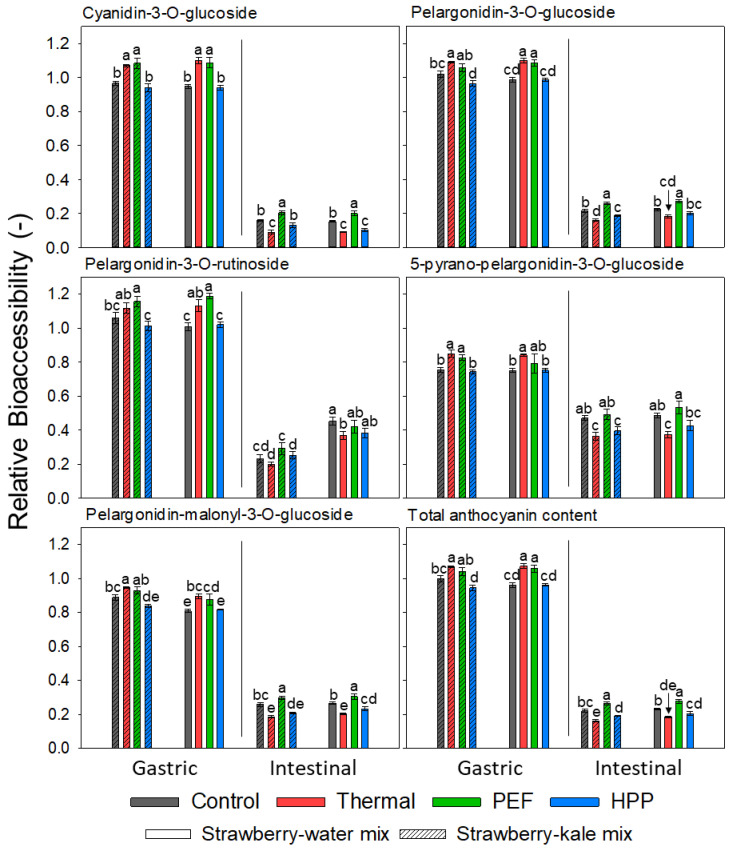
Bioaccessibility (compared to the initial content of the extracted untreated strawberry) of the anthocyanins after processing. Different letters indicate significant differences per anthocyanin and per bioaccessibility (gastric and intestinal) (*p* < 0.05, n = 4).

**Figure 8:**
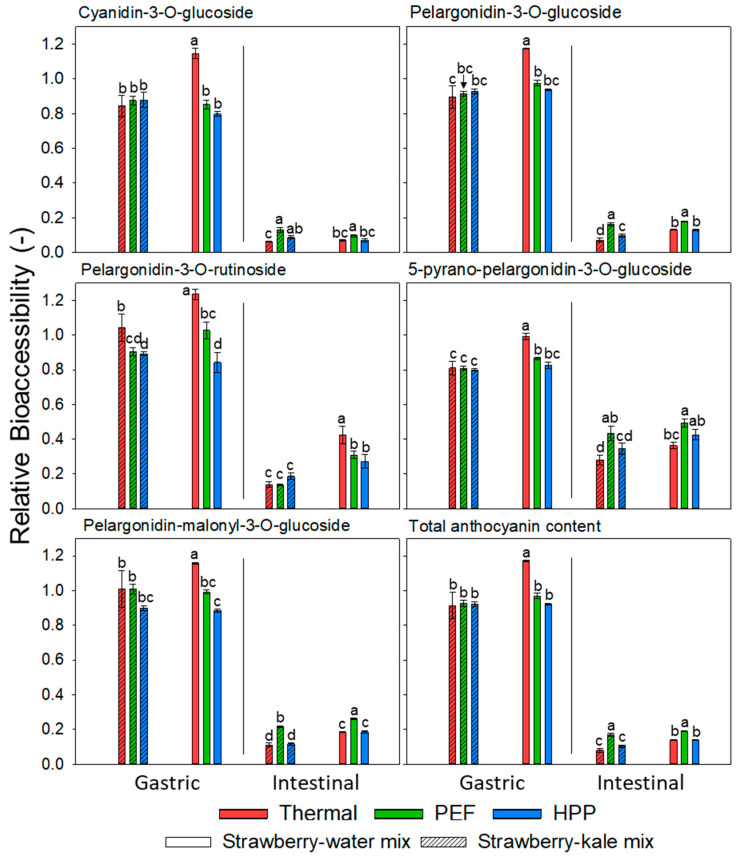
Bioaccessibility (compared to the initial content of extracted untreated strawberry at day 42) of the anthocyanins after storage. Different letters indicate significant differences per anthocyanin and per bioaccessibility (gastric and intestinal) (*p* < 0.05, n = 4).

The anthocyanins were stable during gastric digestion in all the investigated samples, followed by a strongly pronounced decrease during the intestinal digestion (Figure 7 and Figure 8). As the pH value decreased during gastric digestion, the anthocyanin concentrations were relatively stable and, sometimes, even increased due to the augmented presence of the flavylium cation of anthocyanins, which is the most stable form [82]. During the intestinal digestion in mild alkaline conditions, anthocyanins are considered as highly unstable [83,84]. This leads to the degradation of anthocyanins under such conditions and the formation of new components with altered chemical properties and bioaccessibility.

For the differently processed systems, the thermally and PEF-treated samples exhibited the highest gastric BA and the untreated and HPP-treated samples the lowest, regardless of the formulation. The formulation did not significantly influence the gastric BA, except for the thermally treated samples at the end of the storage. A thermally treated strawberry–water mix showed significantly higher gastric anthocyanin BA compared to the strawberry–kale mix. This is also well-reflected in the stability kinetics of the respective samples, which showed a maximum retention for the thermally treated strawberry-water mix and very low stability in the strawberry-kale mix also, compared to the other processing technologies. 

The intestinal BA of the total anthocyanins was in the range of 16–28% after processing and was further significantly reduced during storage to 8–19% (when calculated relatively to the strawberry–water sample at day 42). This might be due to a reduction of the bioaccessible contents after storage due to the interactions or polymerization or due to an enhanced extractability and, thus, a relatively increased absolute concentration of anthocyanins in the strawberry-water sample after storage. However, the intestinal BA can be assessed as comparably low, as previously reported [37]. However, minor differences as a function of the formulation and processing were observed. The intestinal BA of the total anthocyanins was slightly reduced for the strawberry–kale mix compared to the strawberry-water mix, yet was only significant after storage of 42 days, suggesting a tendency towards reduced intestinal anthocyanin BA in the selected more complex food products, possibly due to interactions with compounds from the kale matrix. The thermal processing resulted in an overall significantly reduced intestinal BA, yet the differences were limited, especially compared to HPP. Neither the gastric nor intestinal BA were significantly influenced by storage, though the absolute amount of bioaccessible anthocyanins was higher in the samples directly after processing. 

Previous reports showed that the limiting factor for the intestinal bioaccessibility of anthocyanins is the alkaline conditions during intestinal digestion and, to a lesser extent, other effects such as the matrix (level of bioencapsulation) and storage [35]. However, some studies observed the influence of the food matrix on the stability and bioaccessibility of anthocyanins. For example, in chokeberry juice, the polyphenols were less stable compared to the pure components in the model systems [83]. The combination of anthocyanin-rich juices with other food products, such as dairy, egg or soymilk, was also investigated. The results varied—some observed a decrease in the bioaccessibility of some anthocyanins in the mixed system, while others reported a protective effect of the food matrix [39,78,84,85]. Thus, the effects of the formulation or co-digestion on the bioaccessibility of anthocyanins seem to largely depend on the type of product and present compounds that anthocyanins are interacting with. Furthermore, the analytical method and the representation of BA (absolute or relative) can significantly influence the results. To the best of our knowledge, no previous studies are investigating the effect of storage on in-vitro bioaccessibility. In this study, no effect of storage on the BA was detected. 

Furthermore, the anthocyanin structures can influence their gastric and, especially, intestinal BA. Gastric BA was slightly but significantly higher for PG and PR and significantly lower for PPG. However, the intestinal BA was significantly higher for PPG, followed by PR, then PMG, then PG and, finally, CG. Pyrano-anthocyanins, such as PPG, were formed from monomeric and acylated anthocyanins with low molecular weight components and reported to be more stable compared to other anthocyanins, especially in mild alkaline conditions [78,86]. 

## 4. Conclusions

This study investigated the stability and relative bioaccessibility of anthocyanins in strawberry puree during refrigerated storage as influenced by processing and formulation with kale juice. 

The degradation of the strawberry anthocyanins during storage followed first-order kinetics, influenced by the juice system, preservation technology and anthocyanin structure. The formulation, including kale, showed a major effect, accelerating the degradation of anthocyanins during storage, possibly due to the high enzyme activity in the strawberry–kale mix. The anthocyanin bioaccessibility after the gastric digestion phase was, overall, high and sometimes increased (relative values > 1), especially for pelargonidin-3-O-glucoside, cyanidin-3-O-glucoside and pelargonidin-3-O-rutinoside being more pronounced after the thermal and PEF treatments, irrespective of the formulation and storage. The anthocyanin bioaccessibility after intestinal digestion was, overall, low, likely due to instability at a neutral pH, and even more pronounced for the strawberry-kale mix compared to the strawberry-water mix and the thermally treated samples. The intestinal bioaccessibility was highest for 5-pyrano-pelargonidin-3-O-glucoside due to higher stability at mild alkaline conditions and lowest for pelargonidin-3-O-glucoside and cyanidin-3-O-glucoside. However, to verify the outcome of the in-vitro study, it is important to measure the impact in-vivo. In conclusion, this research reports evidence that the processing, formulation and anthocyanin structure strongly affect both the stability and bioaccessibility of anthocyanins during storage. Thus, when aiming at the development of new products with improved nutritional values derived from anthocyanins, the matrix interactions, as well as stability with different processing technologies, should be considered. For the enhanced mechanistic understanding of the specific effects of complex matrices, together with alternative processing on the stability of anthocyanins, model studies applying the named processing technologies should be conducted.

## Figures and Tables

**Table 1:** Total soluble solids, protein contents and sugar levels of the untreated juice systems.

Parameter	Strawberry-Kale Mix	Strawberry-Water	Kale-Water
Total soluble solids (°Brix)	6.78 ± 0.24	4.60 ± 0.04	2.71 ± 0.08
Protein content (g/100 g)	1.34 ± 0.08	0.36 ± 0.03	1.03 ± 0.04
Sugar (g/100 g)	3.77 ± 0.04	3.02 ± 0.01	0.67 ± 0.02

**Table 2:** Ascorbic acid/dehydroascorbic acid concentrations (mg/100 g) in the strawberry–kale mix and strawberry–water mix directly after processing (day 0) and after the storage (day 42) (n = 2). Different letters indicate significant differences per column. Kale samples exhibited values below the detection limit (1 mg/100 g) and are not reported.

		After Processing (Day 0)	After Storage (Day 42)
Strawberry–kale mix	Control	4.94 ± 0.23 ^bc^	1.16 ± 0.01 ^d^
	Thermal	5.70 ± 0.11 ^a^	2.00 ± 0.00 ^a^
	PEF	5.08 ± 0.23 ^abc^	1.55 ± 0.13 ^bc^
	HPP	4.33 ± 0.04 ^cd^	1.35 ± 0.01 ^cd^
Strawberry–water mix	Control	4.92 ± 0.45 ^bc^	1.54 ± 0.04 ^bc^
	Thermal	5.33 ± 0.03 ^ab^	1.84 ± 0.13 ^ab^
	PEF	4.96 ± 0.19 ^bc^	1.53 ± 0.10 ^bc^
	HPP	4.18 ± 0.17 ^d^	1.45 ± 0.12 ^cd^

PEF: pulsed electric fields and HPP: high-pressure processing.

**Table 3:** Color differences (Δ*E*) compared to the respective untreated systems directly after processing. Different letters indicate significant differences for each compound (*p* < 0.05) (n = 3).

ΔE	Thermal	PEF	HPP
**Strawberry–water mix**	4.04 ± 0.04 ^b^	4.68 ± 0.01 ^a^	0.79 ± 0.06 ^c^
**Strawberry–kale mix**	8.22 ± 0.09 ^a^	7.67 ± 0.09 ^b^	0.86 ± 0.06 ^c^
**Kale–water mix**	5.03 ± 0.25 ^a^	3.90 ± 0.23 ^b^	0.61 ± 0.30 ^c^

**Table 4:** Color difference (ΔE) compared to the respective sample directly after processing along with storage for the strawberry–kale mix. Different letters indicate significant differences during storage per row (*p* < 0.05) (n = 3).

	Day 3	Day 7	Day 14	Day 21	Day 28	Day 42
**Control**	1.31 ± 0.19 ^f^	2.23 ± 0.12 ^e^	3.03 ± 0.07 ^d^	3.66 ± 0.02 ^c^	4.40 ± 0.01 ^b^	5.75 ± 0.04 ^a^
**Thermal**	0.63 ± 0.05 ^c^	1.71 ± 0.05 ^a^	2.09 ± 0.43 ^a^	2.07 ± 0.39 ^a^	1.81 ± 0.04 ^a^	1.04 ± 0.12 ^b^
**PEF**	1.17 ± 0.06 ^d^	4.33 ± 0.04 ^a^	4.31 ± 0.16 ^ab^	4.05 ± 0.04 ^b^	4.05 ± 0.07 ^b^	3.04 ± 0.05 ^c^
**HPP**	0.76 ± 0.08 ^d^	1.39 ± 0.20 ^c^	2.33 ± 0.19 ^b^	2.40 ± 0.14 ^b^	2.59 ± 0.02 ^b^	3.53 ± 0.05 ^a^

**Table 5:** Degradation rate constant *k* (× 10^−2^) of the major strawberry anthocyanins during storage fitting to the first-order kinetic models (n = 3). The coefficient of determination R² was above 0.9 for all samples. Capital letters indicate significant differences per row (anthocyanin), and small letters indicate significant differences per column (sample formulation and processing) (*p* < 0.05).

Formulation	ProCessing	Cyanidin-3-O-Glucoside	Pelargonidin-3-O-Glucoside	Pelargonidin-3-O-Rutinoside	5-Pyrano-Pelargonidin-3-O-Glucoside	Pelargonidin-3-O-Malonyl-Glucoside	Total AnthocyAnin Content (Sum of AnThocyanins)	Monomeric AnThocyanin Content
Strawberry–kale mix	Control	4.40 ± 0.36 ^aB^	3.64 ± 0.16 ^aC^	3.56 ± 0.04 ^aC^	2.45 ± 0.14 ^aD^	4.47 ± 0.18 ^aB^	3.71 ± 0.09 ^a^	2.86 ± 0.03 ^a^
	Thermal	3.18 ± 0.02 ^bA^	2.67 ± 0.01 ^bBC^	1.96 ± 0.02 ^bD^	1.86 ± 0.03 ^bD^	2.81 ± 0.01 ^bB^	2.69 ± 0.01 ^b^	2.44 ± 0.06 ^b^
	PEF	2.83 ± 0.21 ^bA^	2.42 ± 0.27 ^bcA^	1.71 ± 0.21 ^cB^	1.57 ± 0.24 ^bcB^	2.64 ± 0.19 ^bA^	2.22 ± 0.27 ^c^	2.20 ± 0.08 ^c^
	HPP	2.77 ± 0.06 ^bB^	2.21 ± 0.04 ^cC^	1.75 ± 0.03 ^bcD^	1.51 ± 0.05 ^cdE^	2.86 ± 0.03 ^bB^	2.29 ± 0.04 ^c^	1.77 ± 0.05 ^d^
Strawberry–water	Control	1.89 ± 0.05 ^cB^	1.17 ± 0.02 ^dC^	1.10 ± 0.02 ^dC^	1.14 ± 0.05 ^eC^	1.87 ± 0.03 ^cB^	1.28 ± 0.03 ^d^	1.34 ± 0.12 ^e^
	Thermal	1.35 ± 0.03 ^dA^	0.98 ± 0.06 ^dB^	0.61 ± 0.06 ^fC^	0.76 ± 0.02 ^fC^	1.03 ± 0.06 ^eB^	1.00 ± 0.06 ^d^	0.65 ± 0.04 ^f^
	PEF	1.75 ± 0.04 ^cdA^	1.13 ± 0.03 ^dCD^	0.86 ± 0.03 ^deE^	1.01 ± 0.05 ^efD^	1.30 ± 0.04 ^deB^	1.18 ± 0.03 ^d^	1.16 ± 0.12 ^e^
	HPP	1.71 ± 0.06 ^cdA^	1.11 ± 0.05 ^dCD^	0.86 ± 0.04 ^eD^	1.22 ± 0.18 ^deBC^	1.47 ± 0.04 ^dAB^	1.18 ± 0.04 ^d^	1.19 ± 0.04 ^e^

**Table 6:** Correlation between color a* and the total anthocyanin content during storage.

Formulation	Processing	Pearson Correlation
Strawberry-water	Control	0.782 (<0.05)
	Thermal	0.421 (>0.05)
	PEF	0.528 (<0.05)
	HPP	0.647 (<0.05)
Strawberry-kale mix	Control	−0.767 (<0.05)
	Thermal	−0.236 (>0.05)
	PEF	−0.640 (<0.05)
	HPP	−0.956 (<0.05)
